# Human Mesenchymal Stromal Cells Transplantation May Enhance or Inhibit 4T1 Murine Breast Adenocarcinoma through Different Approaches

**DOI:** 10.1155/2015/796215

**Published:** 2015-04-27

**Authors:** T. Jazedje, A. L. Ribeiro, M. Pellati, H. M. de Siqueira Bueno, G. Nagata, M. Trierveiler, E. G. Rodrigues, M. Zatz

**Affiliations:** ^1^Human Genome and Stem-Cell Center (HUG-CELL), Institute of Bioscience, University of São Paulo, 05508-090 São Paulo, SP, Brazil; ^2^Laboratory of Cancer Immunobiology, Department of Microbiology, Immunology and Parasitology, Paulista School of Medicine, Federal University of São Paulo (EPM-UNIFESP), 04023-062 São Paulo, SP, Brazil; ^3^Stomatology Department, Faculty of Dentistry, University of São Paulo, 05508-000 São Paulo, SP, Brazil

## Abstract

The use of Mesenchymal Stromal Cells (MSCs) aiming to treat cancer has shown very contradictory results. In an attempt to clarify the contradictory results reported in the literature and the possible role of human fallopian tube Mesenchymal Stromal Cells (htMSCs) against breast cancer, the aim of this study was to evaluate the clinical effect of htMSCs in murine mammary adenocarcinoma using two different approaches: (1) coinjections of htMSCs and 4T1 murine tumor cell lineage and (2) injections of htMSCs in mice at the initial stage of mammary adenocarcinoma development. Coinjected animals had a more severe course of the disease and a reduced survival, while tumor-bearing animals treated with 2 intraperitoneal injections of 10^6^ htMSCs showed significantly reduced tumor growth and increased lifespan as compared with control animals. Coculture of htMSCs and 4T1 tumor cells revealed an increase in IL-8 and MCP-1 and decreased VEGF production. For the first time, we show that MSCs isolated from a single source and donor when injected in the same animal model and tumor can lead to opposite results depending on the experimental protocol. Also, our results demonstrated that htMSCs can have an inhibitory effect on the development of murine mammary adenocarcinoma.

## 1. Introduction

Mesenchymal Stromal Cells (MSCs) are undifferentiated multipotent cells with potential for self-renewal and differentiation into several distinct cell lineages [1]. They are composed of a heterogeneous population of cells, constituting a reservoir within the connective tissue of most organs involved in the maintenance and repair of tissues throughout the course of life. MSCs present a similar profile of cell surface receptor expression, although they are defined by their functional properties rather than by marker expression.

MSCs can be isolated from different tissues [2–6]. We have previously described the presence of MSCs in human fallopian tube (human tube Mesenchymal Stromal Cells—htMSCs) that were able to differentiate into cartilage, muscle, bone, and adipose cell lineages* in vitro *[6]. Moreover, htMSCs were able to enhance bone maturation* in vivo* in a xenotransplanted model, suggesting that in the future they might be used to treat bone diseases, such as osteoporosis [7]. Breast cancer, the leading form of cancer in women and the second leading cause of cancer mortality worldwide, is a very complex disease and treatment protocols are continually changing [8].

Previous studies aiming to analyze the clinical effect of MSCs in cancer have shown very discrepant results, enhancing [9–11] or inhibiting tumor growth [12–14] in animal models which were injected with different MSCs and with different tumor cell lines. Klopp and colleagues [15] published an important review on the discrepant results warning that experiments made with different methodologies cannot be compared. For example, different protocols were reported for cell-injections (coinjection, systemically, subcutaneously, or intraperitoneally), number and origin (human or murine) of injected MSCs, and injection's schedule of MSCs in each model (before, during, or after the establishment of primary tumor).

One of the best known models for breast cancer studies is the 4T1 murine mammary tumor cell line. Originally isolated by Miller et al. [16], the 4T1 cell line inoculated at the mammary fat pad presents a high tendency to metastasize to several organs such as lungs, liver, brain, and bone, which are also involved in human breast cancer [17, 18].

Muehlberg et al. [19] showed that murine adipocyte stem cells (mASCs) promote tumor growth* in vivo* when coinjected with 4T1 mammospheres or when systemically injected 12 hours after 4T1 local injection. Altman et al. [20] also showed that human ASCs injected intravenously or subcutaneously coinjected with 4T1 cell line are directed to the tumor site, increasing its volume. But the observed results were significant only in the subcutaneously coinjected group.

In an attempt to clarify these controversial results, the aim of this study was to assess the effect of htMSCs on 4T1 murine breast carcinoma development, using two different approaches: (1) coinjection of htMSCs and tumor cells and (2) injection of htMSCs in tumor-bearing animals.

## 2. Materials and Methods

### 2.1. Human Tube MSCs Culture Establishment

Four human fallopian tubes (hFTs) were obtained from hysterectomy or tubal ligation/resection samples collected during the proliferative phase from fertile women. Informed consent was obtained from each patient and approval granted by the Biosciences Institute Ethics Committee of the University of São Paulo.

Cell lines were obtained as described previously [6], with modifications. hFTs samples were washed twice in phosphate saline buffer (PBS, Life Technologies, Carlsbad, CA), finely minced with a scalpel, and put inside a 50 mL conical tube. Then, 5 mL of 0.1% collagenase (Sigma-Aldrich) diluted in PBS was added and samples were incubated for 15 minutes, at 37°C, in a water bath. After the first incubation, 5 mL of pure DMEM/F-12 (Life Technologies) was added and gently mixed. Shortly thereafter, 10 mL of pure TripLE Express (Invitrogen, Carlsbad, CA) was added, gently mixed, and incubated for 15 minutes, at 37°C, in a water bath. Subsequently, supernatant was removed with a sterile Pasteur pipette; cells were washed once with 20 mL of DMEM/F-12 supplemented with 10% fetal bovine serum (FBS, Life Technologies) and pelleted by centrifugation at 400 g for 5 minutes at room temperature. Cells were then plated in plastic flasks (25 cm^2^, Corning, New York, USA) in DMEM/F-12 Media-GlutaMAX-I (5 mL) supplemented with 10% FBS, 100 IU/mL penicillin, 100 IU/mL streptomycin, and 1% nonessential amino acids solution (all Invitrogen) and maintained in a humidified atmosphere of 5% CO_2_ in air at 37°C. The culture medium was routinely replaced twice a week thereafter.

In the third passage, htMSCs were characterized by their differentiation potential and superficial markers (flow cytometry), as described below.

One lineage was randomly chosen for* in vivo *experiments. The other 3 lineages were analyzed* in vitro*.

### 2.2. MSCs Characterization

To evaluate the properties of htMSCs differentiation, adherent cells underwent* in vitro* adipogenic, chondrogenic, and osteogenic differentiation using Life Technologies Stem Prodifferentiation medium kits (A1007101, A1007001, and A1007201), as indicated by the manufacturer. Flow cytometric analysis was provided for antihuman antibodies CD14 (VMRD Inc., Pullman, WA), CD29-PE-Cy5, CD31-PE, CD44-FITC, CD45-FITC, CD73-PE, CD90-R-PE, human leukocyte antigens- (HLA-) ABCFITC and HLA-DR-R-PE (Becton Dickinson), and SH4 (kindly provided by Dr. Irina Kerkis, Butantan Institute, São Paulo, Brazil). Unconjugated markers were reacted with anti-mouse PE secondary antibody (Guava Technologies). All methods were described before [6, 7].

### 2.3. Tumor Cell Line

Murine mammary adenocarcinoma cells (4T1 cell line), obtained from American Type Culture Collection (ATCC, Manassas, VA, USA), were expanded in RPMI-1640 medium (pH 7.2), supplemented with 10% FBS, 10 mM HEPES [N-(2-hydroxyethyl) piperazine-N′-(2-ethanesulfonic acid)], and 24 mM NaHCO_3_ (all from Life Technologies).

### 2.4. Animals

Fifty-one 8-week-old immunocompetent BALB/c female mice, from Inbred Mice Bioterium of Institute of Biomedical Sciences of Universidade de São Paulo (ICB/USP) and from Centre for Development of Experimental Models for Medicine and Biology (CEDEME/UNIFESP), were used. This research, which involves the use of murine tumor cells and human stromal cells in murine animal models, was approved by the Research Ethics Committee of the Federal University of São Paulo. For the experimental groups, the animals were divided into subgroups of 6 or 7 animals.

### 2.5. *In Vivo* Experimental Design

#### 2.5.1. Coinjection of MSCs and 4T1 Tumor Cell Lineage

**Figure 1:**
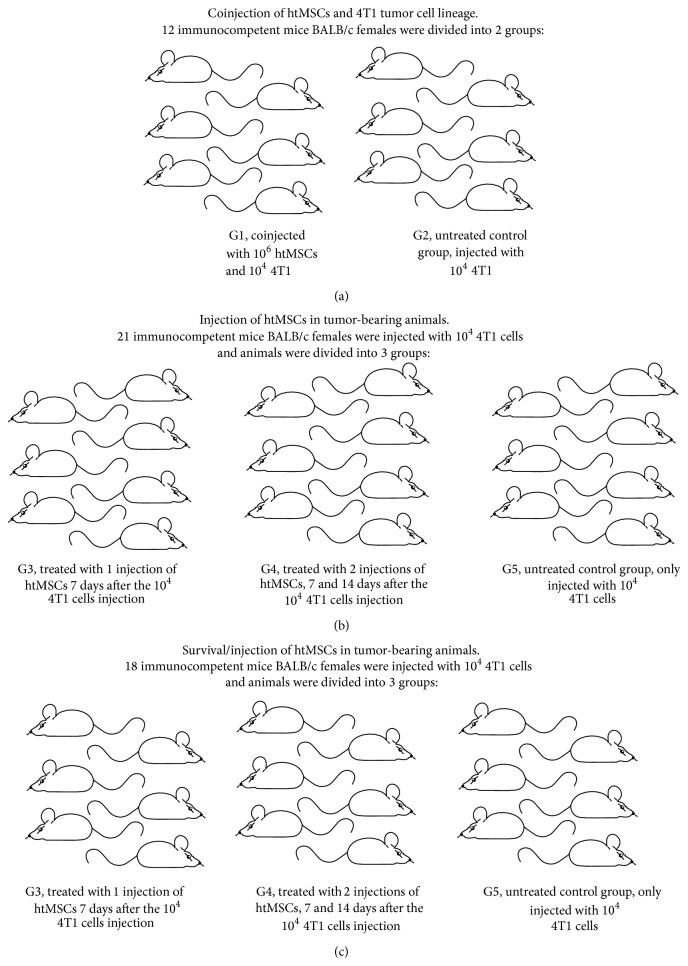
Experimental design, where (a) represents the coinjected group; (b) and (c) represent tumor-bearing animals injected with htMSCs.

For this experiment, 12 BALB/c mice were divided into 2 groups of 6 animals: G1, coinjected in the mammary fat pad with 10^6^ htMSCs and 10^4^ 4T1, and G2, untreated control group, injected in the mammary fat pad with 10^4^ 4T1 (Figure 1(a)).

#### 2.5.2. Injection of htMSCs in Tumor-Bearing Mice

For this experiment, 21 BALB/c mice were firstly injected with 10^4^ 4T1 cells into the mammary fat pad and afterwards the animals were divided into 3 groups of 7 animals per group: G3, treated with 1 intraperitoneal injection of 10^6^ htMSCs, 7 days after the inoculation of 4T1 cells; G4, treated with 2 intraperitoneal injections of 10^6^ htMSCs, 7 and 14 days after the inoculation of 4T1 cells; G5, untreated control group, only injected with 4T1 cells (Figure 1(b)).

#### 2.5.3. Survival

For survival analysis, 18 BALB/c mice were divided into 3 groups of 6 animals, treated as described in Section 2.5.2 and monitored daily until natural death (Figure 1(c)).

The protocol for tumor 4T1 cells inoculation (10^4^ cells injected into the mammary fat pad) was previously standardized (data not shown). In these conditions, primary tumors were visible in about 7 days. The MSCs dose of 10^6^ cells apparently was well tolerated after intraperitoneal or intravenous injections in mice, with no visible changes in animals.

### 2.6. Primary Tumor Growth

During the* in vivo* experiments and postmortem, primary tumor volumes were measured with a mechanical caliper every three days, and the tumor volume was calculated using the formula [(higher  value)(smaller  value)^2^] × 0.52.

### 2.7. Postmortem Animals Examination

Three coinjected animals were analyzed right after natural death. The remaining mice were analyzed right after euthanasia in the CO_2_ gas chamber. Primary tumors were collected and the presence of intraperitoneal metastatic tumors was registered by digital images.

### 2.8. Tissues Histology

Two primary tumors and lungs from each experimental group were fixed in 10% formalin (diluted in 1X PBS) for one week at room temperature and paraffin-embedded. For histological analysis, slides (5 *μ*m thick) were cut and dyed with Hematoxylin-Eosin. Additionally, primary tumors were analyzed for the presence of human cells through the analysis of the specific human nuclei lamin A/C (anti-lamin A + C, Abcam Inc., Cambridge, MA, USA).

### 2.9. Pulmonary Nodules and Inflammation Analysis

For this analysis, the lungs of all animals were removed and dyed with Bouin's Solution. After 48 h, Bouin's Solution was removed and replaced by 10% formalin (diluted in 1X PBS). Digital images were obtained from each organ and pulmonary metastases/nodules were counted in a stereomicroscope (Nikon, Tokyo, Japan). Afterwards, two lungs from each experimental group were paraffin-embedded for histological analysis (Hematoxylin-Eosin). The levels of pulmonary inflammation and tumor tissues (metastasis) were analyzed measuring the free area, that is, the tissue-free space, of each lung (tool available in the software NIS Elements Nikon AR).

### 2.10. Immunohistochemistry

For immunohistochemistry analysis, 3 *μ*m sections of primary tumor specimens were deparaffinized, rehydrated, and incubated in 6% aqueous hydrogen peroxide for 30 min to quench endogenous peroxidase activity. The slides were heated to 95°C for 45 min in EDTA buffer for antigen retrieval and treated with 0.5% pepsin, pH 1.8 for 30 min at 37°C. The sections were incubated with a human specific anti-lamin A + C antibody (ab108595, Abcam, Cambridge, UK). ENVISION HRP system (Dako, Carpinteria, CA, USA) was used to detect the nuclear lamin proteins of the htMSCs. Samples were lightly counterstained with Mayer's hematoxylin, dehydrated, and mounted with glass coverslips and xylene-based mounting medium. Nonimmune serum was used as negative control, and human-origin cartilage micromass, originated by htMSCs chondrogenic differentiation* in vitro* [6], was used as positive control.

### 2.11. Identification of Cytokines Released by htMSCs* In Vitro*

Aiming to verify the production of cytokines released by htMSCs when they are in the tumor microenvironment, we cultivated htMSCs with 4T1 tumor cells* in vitro* at the ratio 1 : 1 (2 × 10^5^ cells) in 6-well plates (3 mL of culture media per well). We used media DMEM/F-12 supplemented with 20% of FBS and 100 IU/mL penicillin and 100 IU/mL streptomycin (all Life Technologies). Cells were physically separated by culture inserts (0.4 *μ*m PET, Millipore, Darmstadt, Germany) and maintained in a humidified atmosphere of 5% CO_2_ at 37°C for 48 h.

The Bio-Plex Pro Human Cytokine 27-Plex Immunoassay Panel (Bio-Rad, Hercules, CA, USA, number M50-0KCAF0Y) includes 27 magnetic bead-based assays to measure FGF basic, eotaxin, G-CSF, GM-CSF, IFN-*γ*, IL-1*β*, IL-1ra, IL-2, IL-4, IL-5, IL-6, IL-7, IL-8, IL-9, IL-10, IL-12 (p70), IL-13, IL-15, IL-17, IP-10, MCP-s (MCAF), MIP-1alpha, MIP-1beta, PDGF-BB, RANTES, TNF-alpha, and VEGF. Supernatant was harvested and processed according to the Bio-Assays-Plex Pro manufacturer's instructions. For this experiment, we used three lineages of different htMSCs, each one in triplicate.

### 2.12. Statistical Analyses

Statistical analyses were done by ANOVA test with Tukey's test track (by Microsoft Excel 2010) and by the Software Prism 5 for the survival analysis.

## 3. Results

### 3.1. htMSCs Characterization

htMSCs used in this experiment differentiated in adipogenic, chondrogenic, and osteogenic tissues* in vitro* and presented as well the expected profile of surface markers by cytometry, as described before [6, 7] (data not shown).

### 3.2. Coinjection of htMSCs and 4T1 Tumor Cell Lineage

#### 3.2.1. Tumor Growth and Inflammation Analysis

**Figure 2:**
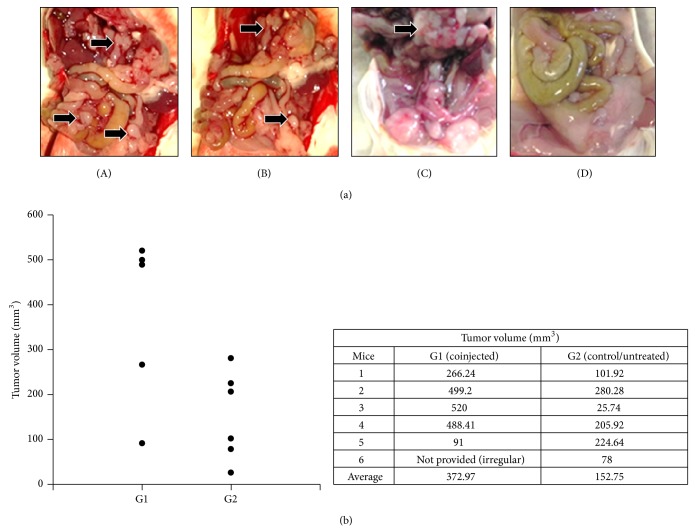
Coinjected group necropsy and tumor growth analysis. (a) Abdominal view of coinjected animals (A, B, and C) and untreated control (D), on day 15. Arrows indicate probable tumor nodules. One representative animal of each group is shown. (b) Primary tumor volume at death (day 15), showing that coinjected animals (G1) presented, on average, primary tumor volumes about 2.4x higher than the untreated group (G2). Animals are represented individually.

All animals developed primary tumors. However, some coinjected animals (G1) survived only 15 days, and the necropsies realized in all animals at day 15 showed many tumor masses in the abdominal and thoracic region of G1 group, while untreated animals (G2) presented only primary tumor growth and no visible nodules in the abdominal/thoracic region (Figure 2(a)). Furthermore, the primary tumor volume was significantly increased in 4 of 6 animals of the coinjected group (Figure 2(b)).

**Figure 3:**
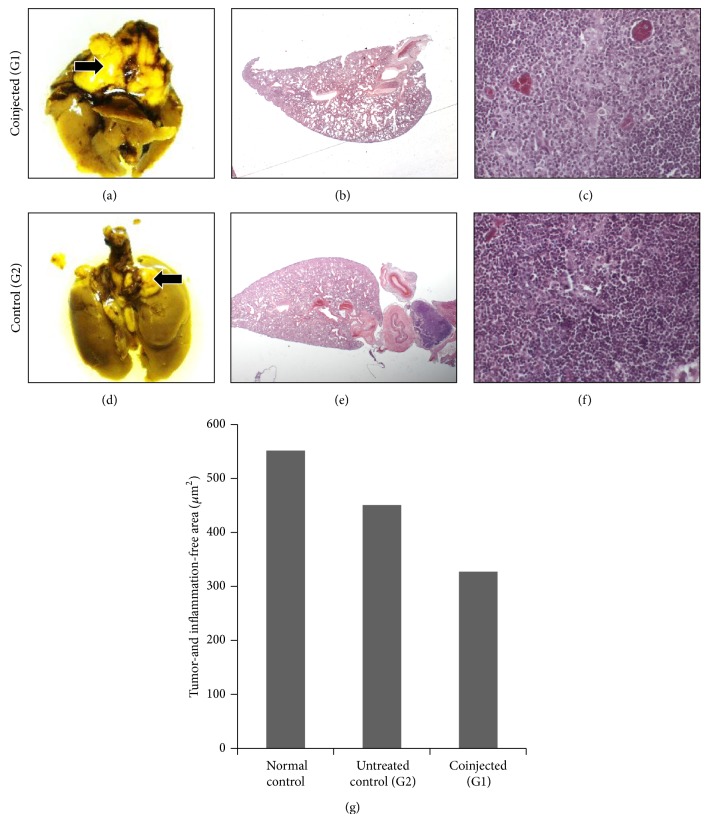
Tumor analysis of G1 and G2 groups. ((a) and (d)) Lungs macroscopic view; ((b) and (e)) lungs microscopic view (2.5x); ((c) and (f)) primary tumors microscopic view (40x). One representative animal of each group is shown. (g) Tissue-free measurement in lungs, showing that coinjected animals presented an increase in inflammation areas in the lungs, despite not having died from respiratory insufficiency. Arrows indicate possible tumor masses near trachea in (a) and (d).

Macroscopic lung analysis showed preserved organs but possible tumor masses near the trachea in all coinjected animals. Microscopic analysis of the lungs showed no visible tumor nodules and primary tumors with similar histology in both groups. Although no differences in lungs were evident between the groups in macro- and microscope analysis, when the size of tissue-free areas in the lungs was compared, a reduction of 40% in the coinjected group was observed (Figure 3). These results suggest that htMSCs coinjected with 4T1 breast carcinoma cells exacerbate primary tumor growth, reduce the inflammation-free area of the lungs, and facilitate abdominal metastasis development.

### 3.3. Injection of htMSCs in Tumor-Bearing Mice

**Figure 4:**
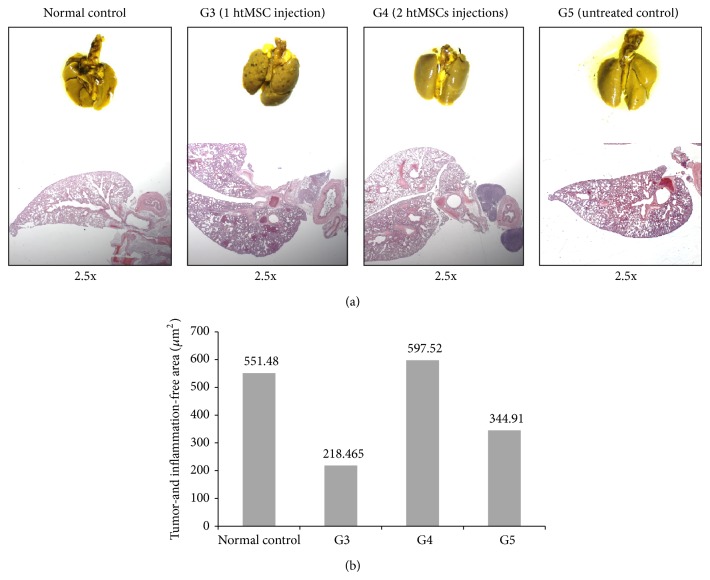
Inoculation of htMSCs in tumor-bearing mice. (a) Images show G3, G4, and G5 lungs (macroscopically and microscopically), in comparison with normal control (normal mice lung) 20 days after tumor cell inoculation. One representative animal of each group is shown. (b) Pulmonary tissue-free measurements, showing that lungs were preserved in G4 group (2 htMSCs injections), similar to normal controls, represented as tumor- and inflammation-free area.

All groups (G3, G4, and G5) were analyzed 20 days after tumor cells inoculation. None or just few abdominal nodules were found in some animals of the 3 groups, but no lung metastatic nodules were macroscopically visible in any group (Figure 4). Group G4, treated with 2 injections of htMSCs, showed a lower number of microscopic pulmonary nodules on the 20th day when compared to group G3 and the untreated control group (G5), as expected, since the animals were in better physical conditions. But it is important to point that all animals (G3, G4, and G5 groups) presented microscopically visible tumors 20 days after the onset of the experiment. Differences between groups were also evident when the tissue-free lung area was compared, showing that treatment of mice with 2 doses of htMSCs restored the lung areas free of inflammation and metastasis, as compared to normal mice (Figure 4(b)).

### 3.4. Immunohistochemistry

**Figure 5:**
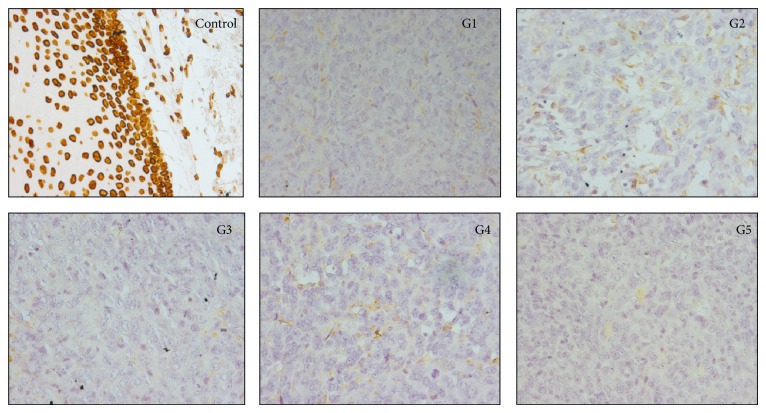
Human nuclei analysis in primary murine breast tumors, using the human specific antibody anti-lamin A + C, in G1 to G5 groups. Primary tumors were collected 15 days after tumor cell inoculation. Positive control (human tissue), showing positive staining. Slide of one representative animal of each group is shown. The antibody did not stain any structure in all primary tumors analyzed.

Human nuclei were found neither in primary tumor (Figure 5) nor in lung metastasis (data not shown) in animals from any group, including the coinjected group G4, 15 days after tumor cell inoculation. This result suggests that tumors were formed exclusively by murine tumor cells, and the injected htMSCs were not present at the tumor microenvironment in the evaluated timepoint.

### 3.5. Survival and Tumor Growth

**Figure 6:**
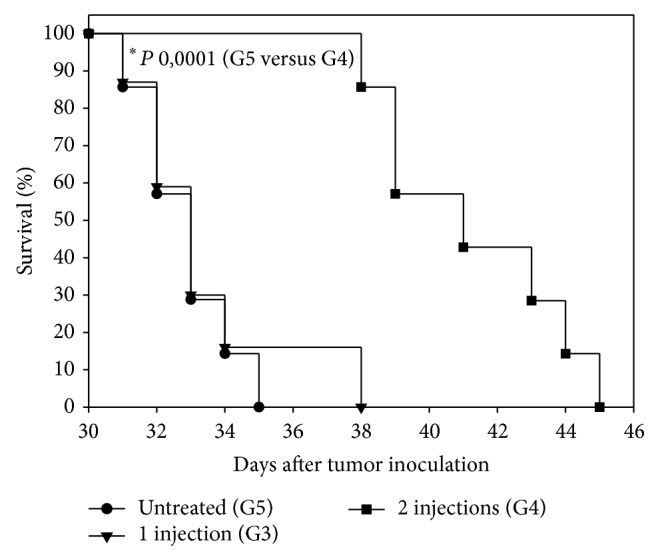
Survival analysis of tumor-bearing mice inoculated intraperitoneally with htMSCs. Animals (six animals per group) treated with 2 htMSCs injections (G4) showed a statistically significant increase in survival compared to untreated animals (control, G5).

**Figure 7:**
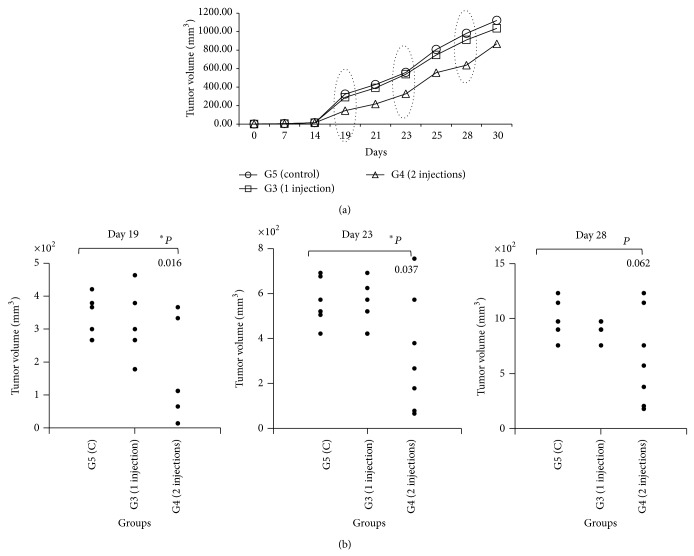
Primary tumor development in tumor-bearing mice inoculated intraperitoneally with htMSCs. (a) The average tumor volume of G3, G4, and G5 groups in each day is represented. (b) Tumor volumes of individual animals on days 19, 23, and 28. Animals injected with 2 doses of htMSCs showed significantly reduced tumor development compared to untreated control until 23 days after tumor inoculation.

Animals in the control group (G5) died from day 30 to day 35. The group treated with only 1 htMSCs injection (G3) started dying on day 31, and at day 37, 85% of animals had already died. On the contrary, only 15% of animals treated with 2 htMSCs injections (G4) had died at day 38, which represent a highly statistically significant difference as compared to the control group (^∗^ *P* value = 0.0001) (Figure 6). Furthermore, 2 htMSCs injections reduced primary tumor volumes in G4 group as compared to the other 2 groups (Figure 7(a)). At least until day 23 after tumor inoculation, average primary tumor volume of the G4 group was significantly reduced compared to untreated control group (G5). Statistically significant differences were lost in later measurements (Figure 7(b)).

### 3.6. Identification of Cytokines Released by htMSCs* In Vitro*

**Figure 8:**
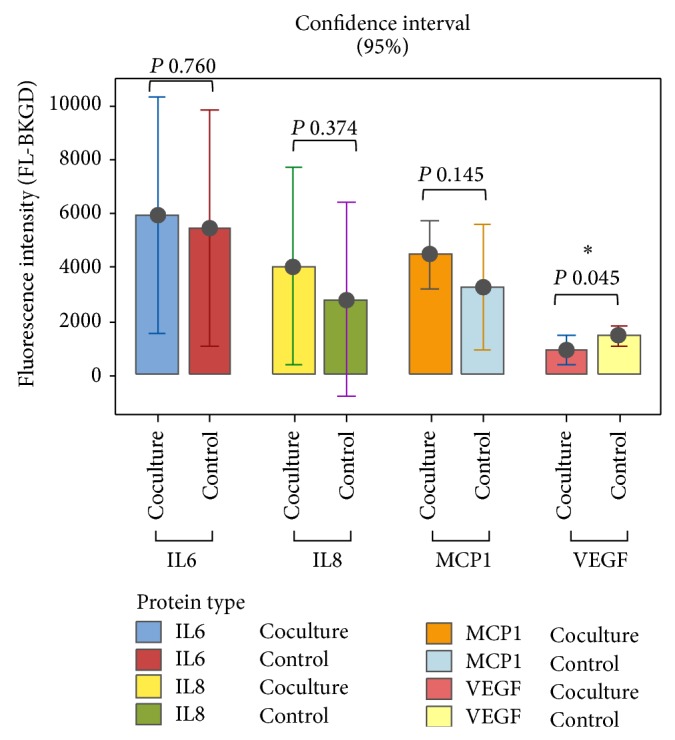
Concentration of cytokines released by htMSCs before (control) and after coculture with murine 4T1 tumor cells (with no direct contact). A small increase (about 9%) of IL6, an important increase of IL-8 and MCP1 (about 45% and 37%, resp.), and a decrease of VEGF expression (about 36%) were observed (*P* = 0.045). All samples were analyzed in triplicate. The results represent the mean and standard deviation of each triplicate.

Murine tumor cells and htMSCs were cultivated separately (controls) in complete medium for 48 h and human cytokines were analyzed in the culture supernatant. As expected, human cytokines were not detected in the control murine cells supernatant. In the culture supernatant of control htMSCs, only 4 cytokines were detected among the 27 analyzed by the assay: IL-6, IL-8, MCP1, and VEGF. After cocultivation with no direct contact of both cell lines (using a transwell), there was a substantial increase (about 48% and 37%, resp.) in the secretion of IL-8 and MCP1. In contrast, a decrease in the levels of VEGF released by htMSCs (about 36%) was observed after cocultivation of cell lines in the described conditions. Due to the intraindividual variation of each htMSC analyzed, only the VEGF showed statistical significance, although a proinflammatory tendency is evident for other expressed cytokines (Il-6, IL-8, and MCP-1) (Figure 8).

## 4. Discussion

Here we show, for the first time, that human MSCs obtained from one single source and cultivated under the same conditions, when injected in animals with the same disease, can produce opposite results depending on the experimental protocol. When we compared the effect of subcutaneous coinjections of htMSCs and tumor cells with intraperitoneal injections of the same htMSC lineage in immunocompetent animals of the same age and background inoculated previously with the same tumor cells, we observed a beneficial effect only in animals in which the tumor was already established before the intraperitoneal htMSCs inoculation. When htMSCs were coinjected subcutaneously with 4T1 cells, we observed an opposite effect, that is, exacerbation on primary and metastatic tumor development.

Several mechanisms have been reported to be responsible for these discrepant observations, such as chemokine signaling, modulation of apoptosis, vascular support, and immune modulation. Suzuki et al. [21] showed that murine bone marrow MSCs increased local neovascularization and tumor growth. It has also been reported that human bone marrow MSCs increased tumor growth and metastasis in murine colon cancer [22].

On the other hand, it is well documented that MSCs release factors with angiogenic and immunomodulatory properties which was observed even in xenotransplantation of human MSCs in animal models [23, 24]. Therefore, in order to verify if the observed results could be related to cytokines and chemokines released by htMSCs in the tumor microenvironment, we performed* in vitro* cocultures of htMSCs and 4T1 cells.

After 48 hours of coculture with no direct contact of htMSCs and 4T1 tumor cells, we observed a significant increase of IL-8 (interleukin-8) and MCP-1 (monocyte chemoattractant protein-1), 44% and 37%, respectively, as well as a decrease (36%) of VEGF (vascular endothelial growth factor). This result shows that unknown factors released by murine tumor cells can regulate the production and secretion of IL-8, MCP-1, and VEGF by htMSCs.

IL-8, alternatively known as CXCL8, is a proinflammatory chemokine highly related to the progression of cancer, since many studies have shown overexpression of IL-8 by tumor cells. It is a chemotactic factor exerting a large migratory stimulus to immune system cells, especially neutrophils. It also determines an increase in the expression of adhesion molecules by endothelial cells [25]. Also, Fujimoto and colleagues [26] showed that MCP-1 induces tumor-associated macrophage infiltration and contributes to tumor progression in immunodeficient mice bearing human breast cancer cells by recruiting monocytes to injury sites, triggering thus a proinflammatory reaction. It has been shown that chemotactic proteins such as MCP-1 and IL-8 promote migration of human MSCs* in vitro* and induce the recruitment of leukocytes to the injured sites [27]. Therefore, the increased expression of IL-8 and MCP-1 we found in the coculture htMSCs/4T1 media suggests that the increased secretion of these molecules at the tumor microenvironment can be related to the increased tumor growth observed when these cells were coinjected* in vivo*. Although we were not able to detect human MSCs at established primary tumor sites, our results suggest that the interaction of these cells during the implantation period of tumor cells after coinjection can facilitate and stimulate tumor growth.

VEGF is a cytokine strongly related to angiogenesis regulated by microenvironmental factors within the tumors, such as hypoxia, free radicals, pH imbalance, and nutrient deficiency. Its expression may be influenced by a number of microenvironmental factors which may play important role in regulating VEGF expression during tumorigenesis [28]. On the contrary, proangiogenic factors can also have an immunosuppressive effect. Vascular endothelial growth factor A (VEGF-A) can induce the accumulation of immature dendritic cells, myeloid-derived suppressor cells, and regulatory T cells and inhibit the migration of T lymphocytes to the tumor. It has been suggested that other proangiogenic factors such as placental growth factor (PlGF) could also participate in tumor-induced immunosuppression [29].

The reduced VEGF secretion by htMSCs in coculture with 4T1 cells suggests that the expression of IL-8 and MCP-1 by htMSCs at the tumor microenvironment after coinjection of mesenchymal and tumor cells strongly regulates the increased primary and metastatic tumor development, by chemoattracting secondary immune cells, with a minor participation of VEGF in these conditions. We hypothesize that the influence of these htMSCs-secreted factors at the beginning of tumor establishment at the mammary fat pad is very important for the exacerbation of tumor growth and metastasis.

Although our results showed that htMSCs were not found at the primary tumor 15 days after tumor cell inoculation, we cannot exclude that these cells were recruited to the tumor site immediately after intraperitoneal inoculation but could not survive long in this murine environment. The production of reduced concentrations of VEGF by htMSCs, leading to a less immunosuppressive tumor environment, in association with the recruitment of immune cells by IL-8 and MCP-1, after tumor cells establishment (first dose) and during tumor development (second dose), could explain the significant tumor growth control induced by the treatment protocol.

Corroborating our hypothesis that the immune system has an important participation in the effects observed after both protocols, it has been shown that another variant that could influence the role of MSCs in tumor development is the use of immunodeficient/immunosuppressed or immunocompetent animal models. According to Barcellos-de-Souza et al. [30], several* in vivo* assays that performed coinjections of MSCs with different types of tumor cells in immunocompromised animals showed an increase in tumor growth. Among them are models of colon cancer, osteosarcoma, ovarian cancer, colorectal cancer, melanoma, lung cancer, gastric cancer, and prostate carcinomas. In opposition, Lu et al. [31] showed an inhibition of ascites formation in an immunocompetent murine model of ascitogenous hepatoma after three injections of murine bone marrow MSCs, zero, three, and ten days after tumor cell inoculation.

Previous studies from our and other groups have shown that MSCs from different sources, such as umbilical cord, dental pulp, and adipose tissue [32–34], may have different clinical effect when injected in animal models for neuromuscular disorders. However, some properties such as immunomodulatory potential are apparently a common characteristic of MSCs [35].

Here, we show that the same MSCs, injected in the same animal model, may lead to opposite results according to the experimental procedure. Our results reinforce that the moment when MSCs reach tumor microenvironment and apparently secrete factors to recruit other immune cells after interaction with tumor cells is crucial for tumor development.

We are not aware of other studies comparing the clinical effects of htMSCs in immunocompetent mice developing a breast adenocarcinoma. Therefore it is very important to verify whether the beneficial effect we observed in delaying tumor growth and increasing the life span of 4T1 breast tumor-bearing immunocompetent mice also occurs with MSCs from other sources. This is particularly relevant since any approach aiming to treat human cancer will be done in patients with established tumors.

## 5. Conclusions

In short, here we show that (1) htMSCs promote and/or accelerate breast adenocarcinoma in immunocompetent mice when coinjected with 4T1 tumor cells; (2) htMSCs can be beneficial to the animals that already have an established breast cancer at initial stages, depending on the dose and the route of administration of the injected htMSCs, decreasing primary and metastatic tumor growth and significantly increasing their survival; (3) repeating these experiments with MSCs from other sources is of utmost importance.
